# Emerging and re-emerging disease threats in the Middle East and North Africa region—One Health approaches and potential strategies

**DOI:** 10.1093/eurpub/ckae122

**Published:** 2025-01-13

**Authors:** Sean V Shadomy, Shahul H Ebrahim, Sarah Anne J Guagliardo, Liliana Sánchez-González, Kinda Zureick, Julie R Sinclair, Dana A Schneider, Allison T Walker, Daniel C Payne, Antonio R Vieira, Kristin Heitzinger, Audrey Lenhart, Lisa P Oakley, Jacob Clemente, Colin Basler, Charles B Beard, Paige A Armstrong, Heather Burke

**Affiliations:** National Center for Emerging and Zoonotic Infectious Diseases, Centers for Disease Control and Prevention, Atlanta, GA, United States; Global Health Center, Centers for Disease Control and Prevention, Atlanta, GA, United States; National Center for Emerging and Zoonotic Infectious Diseases, Centers for Disease Control and Prevention, Atlanta, GA, United States; National Center for Emerging and Zoonotic Infectious Diseases, Centers for Disease Control and Prevention, Atlanta, GA, United States; National Center for Immunizations and Respiratory Diseases, Centers for Disease Control and Prevention, Atlanta, GA, United States; National Center for Immunizations and Respiratory Diseases, Centers for Disease Control and Prevention, Atlanta, GA, United States; National Center for Emerging and Zoonotic Infectious Diseases, Centers for Disease Control and Prevention, Atlanta, GA, United States; National Center for Emerging and Zoonotic Infectious Diseases, Centers for Disease Control and Prevention, Atlanta, GA, United States; National Center for Emerging and Zoonotic Infectious Diseases, Centers for Disease Control and Prevention, Atlanta, GA, United States; National Center for Emerging and Zoonotic Infectious Diseases, Centers for Disease Control and Prevention, Atlanta, GA, United States; National Center for Emerging and Zoonotic Infectious Diseases, Centers for Disease Control and Prevention, Atlanta, GA, United States; National Center for Emerging and Zoonotic Infectious Diseases, Centers for Disease Control and Prevention, Atlanta, GA, United States; National Center for Emerging and Zoonotic Infectious Diseases, Centers for Disease Control and Prevention, Atlanta, GA, United States; National Center for Emerging and Zoonotic Infectious Diseases, Centers for Disease Control and Prevention, Atlanta, GA, United States; National Center for Emerging and Zoonotic Infectious Diseases, Centers for Disease Control and Prevention, Atlanta, GA, United States; National Center for Emerging and Zoonotic Infectious Diseases, Centers for Disease Control and Prevention, Atlanta, GA, United States; National Center for Emerging and Zoonotic Infectious Diseases, Centers for Disease Control and Prevention, Atlanta, GA, United States; Global Health Center, Centers for Disease Control and Prevention, Atlanta, GA, United States

## Emerging and re-emerging threats in Middle East and North Africa

Emerging and re-emerging infectious diseases have no boundaries, and the Middle East and North Africa (MENA) region is no exception (For this discussion the authors define the Middle East and North Africa region as comprised of the following: countries: Afghanistan, Algeria, Bahrain, Djibouti, Egypt, Iran, Iraq, Israel, Jordan, Kuwait, Lebanon, Libya, Morocco, Oman, Pakistan, Palestine, Qatar, Saudi Arabia, Somalia, South Sudan, Sudan, Syria, Tunisia, Türkiye, United Arab Emirates, and Yemen.) [1, 2]. Given the intricate mesh of commerce and livestock trade, mass gathering events, international travel, and population movement throughout the region, emerging health security threats in MENA can have global implications, The emergence of Middle East Respiratory Syndrome coronavirus (MERS-CoV) in Jordan and Saudi Arabia in 2012 elevated MENA as a disease emergence hotspot. The region has seen multiple pathogen emergence events during recent decades. (see Supplementary Box S1).

Vector-borne diseases (VBD) in the region range from parasitic diseases such as malaria and leishmaniasis to arboviruses including *flaviviruses* (e.g. West Nile, dengue, Alkhurma hemorrhagic fever), the *phlebovirus* Rift Valley Fever (RVF), and the *alphavirus* chikungunya [3–5]. A geographically diverse region, MENA countries have many arthropod disease vectors, including multiple species of mosquitoes and sandflies, and while they may be highly focal, increasing rainfall and water ponding can increase their distribution. *Aedes aegypti* and *Aedes albopictus* mosquitoes (vectors of dengue, Zika, chikungunya, and yellow fever viruses) have been detected in several countries. Of particular concern is the establishment of the invasive mosquito *Anopheles stephensi* in the Horn of Africa and Yemen, threatening regional progress toward malaria elimination [5].

In animal health, Foot and Mouth Disease, Peste des Petits Ruminants, and RVF have all re-emerged in MENA. Highly Pathogenic Avian Influenza (H5N1), first reported in Egypt in 2006 and Low Pathogenic Avian Influenza (H9N2) in Morocco in 2016, have been confirmed in wild and farmed birds in multiple countries and with associated zoonotic transmission [6]. Rabies, bovine tuberculosis, leptospirosis, brucellosis, and parasitic zoonoses occur broadly across the region.

## Contributing factors, drivers, and related issues

A variety of drivers and risk factors influence the emergence, geographic spread, and burden of disease threats in MENA (Table 1). The effects of climate merit particular attention as it influences multiple other contributing factors. Climate change impacts emerging diseases pertinent to human and animal health through three interconnected processes: environmental change leading to cross-species transmission, global migration due to forced displacement, and urbanization. Carlson *et al*. predicted 4000 new cross-species transmission events among wildlife by 2070 due to changes in climate and land use, increasing zoonotic pathogen spillover risk in heavily populated regions of Asia and Africa [7]. According to the World Bank, by 2050, climate change could result in the forced displacement of up to 18 million people in North Africa, Eastern Europe, and Central Asia [8].

**Table 1.** Drivers influencing disease emergence in the Middle East and North Africa (MENA) region^a^

*Conflicts*: In 2022, there were 58 conflicts in the region, of which 28 were violent crises and two full-scale wars, disrupting health systems and civilian infrastructure, inflicting numerous casualties, causing mass displacements of people and their animals, and creating multiple refugee crises. *Population displacement*: In 2022, MENA accounted for one-fourth of the global internally displaced persons (16 million), making MENA the region with the second highest concentration of displaced persons following the sub-Saharan Africa region. *High development disparity*: Globally, MENA has the world’s highest variation in GDP and per capita income, life expectancy and human development index. *Climate change*: MENA is world's most vulnerable region to the impacts of climate change, enduring ever-higher temperatures, droughts, floods and other extreme weather events, increased water scarcity, all of which contribute to shifts in vector species and distribution. El Niño has affected 71% of drought years in the southern and southwestern parts of the Arabian Peninsula, and La Niña was linked to 38% of this area's flood years. *Changing land and water use*: Anthropogenic activities create habitats favorable for vectors, and stress on water resources. *High burden of metabolic and other disorders*: The prevalence of diabetes reported in the MENA averaged 12.2% compared to 9.3% globally. Obesity rates among men range from 2% to 55%. High rates of vitamin D deficiency (60%- 80%) and hemoglobinopathies (thalassemia as high as 43%, sickle cell disease, 0.2%–5.8%, sickle cell trait 1.0%–45.8%) also increase the risk for infections. *Population movement across disease hotspot regions*: Foreign workforce accounts for about 80% of GCC workforce, the majority of whom are unskilled laborers originating from South and Southeast Asia. GCC has emerged as a major international airline hub and tourist destination for the region (37% of arrivals in 2022), Asia (28%) and Africa (8%). *Mass gatherings*: MENA is home to the world’s largest annual international mass gathering involving over 180 countries (the Hajj), the largest domestic mass gatherings (Arbaeen), and a continuous year-round religious pilgrimage to these sites, all of which have been associated with disease transmission events. *Changing animal contact patterns*: Changes in consumption patterns such as of unpasteurized milk impacting rates of brucellosis, and increased risk for MERS-CoV outbreaks due to close contact with infected camels or consumption of raw camel products, are examples. *Animal husbandry, legal and illegal movement of live animals*: Livestock contributes significantly to the GDP in many MENA countries (e.g., 40% of GDP in Somalia). Live animals are imported across MENA countries for food, religious sacrifices, sporting events, and as pets. MENA has also become a hotspot for wildlife trafficking. *Sub-optimal human and animal healthcare infrastructure or coverage*: Countries with economic challenges and conflicts can have suboptimal surveillance systems. Developed country health systems may not capture noncitizen health events. *Antimicrobial resistance (AMR)*: In MENA, in 2019, an estimated 255,676 deaths were associated with, and 68,292 deaths attributable to, bacterial AMR. That year, carbapenem-resistant *Acinetobacter* spp. accounted for 70% of patient bloodstream infections, higher than in the United States and European Union.

a References provided in the Supplementary material.

Surveillance and serosurveys in human and animal populations can be restricted or disrupted, limiting the understanding of disease ecology and presence. The paucity of MERS-CoV detections in Saudi Arabia and the occurrence of Crimean Congo Hemorrhagic Fever (CCHF) outbreaks in Iraq during the COVID-19 pandemic demonstrate how shifting priorities can interrupt disease surveillance and control programs [9,10].

## Strategic One Health approaches and solution

The importance of strengthening human and animal health surveillance is underscored in the 2021 WHO Regional Committee for the Eastern Mediterranean endorsement of the strategy for national integrated disease surveillance systems. By 2023, 11 countries were implementing event-based surveillance, exemplified by Qatar's use for early warning for the FIFA World Cup 2022 [11]. Resources supporting coordinated, One Health-based surveillance and information sharing are more readily available. Incorporation of social and news media sources can improve alerting and mapping pathogen spread. Serosurveillance innovations such as multiplex serology, and approaches such as wastewater sampling are opportunities to enhance pathogen detection and monitoring.

Integrating travelers and other mobile populations into public health surveillance is critical to enhance prevention of cross-border disease spread. Many countries focus efforts at official air, water, and ground points of entry (POE), which can detect and respond to overtly ill travelers, but may fail to detect infection in a- or pre-symptomatic travelers or those bypassing official POEs at porous border areas. Broader strategies that address health risks along the continuum of travel from point of origin to destination can include assessment of human and animal population mobility patterns and the connections between geographically separated communities.

Agent and genomic surveillance at POEs and by mass-gathering clinical services can detect infections in travelers and additionally inform emergence trends in their nations of origin. Such efforts were utilized in the USA during COVID-19 [12] and routinely during the Hajj mass gatherings. Countries with significant international connectivity can include the private sector in surveillance, such as travel clinics complementing early detection efforts and the provision of migrant health services. With support from US CDC, countries in MENA are integrating travel medicine into national programs facilitating surveillance in international visitors and connecting to mobile populations.

Strengthening surveillance for animal health and zoonotic threats including on export and import can help protect economies, food safety and security and health security. Multiagent surveillance in imported livestock in Saudi Arabia has demonstrated the feasibility of such approaches. The FAO, UNEP, WHO and WOAH Quadripartite provides guidance and support to strengthen multisectoral preparedness, surveillance, and response; however, these may be hindered by resource limitations, conflict, or lack of political will. One Health collaborations are critical to success, and a key first step can be establishing regional platforms similar to WHO's Global Influenza Surveillance and Response System, or implementing collaborative approaches for specific threats such as the United Against Rabies “Zero by 30” to eliminate human deaths from canine rabies.

While two dengue vaccines (Dengvaxia and Qdenga), one chikungunya vaccine, and several prequalified yellow fever vaccines have been authorized for use in some countries, they are not widely available and may be subject to usage limitations [13, 14]. Integrated Vector Management (IVM), a framework for vector control and surveillance adopted by seven countries in MENA [15], could be expanded to other countries with high VBD burden. With IVM strategies in mind, in 2021 CDC initiated the VecNet program to strengthen regional public health entomology networks.

Campaigns to control zoonotic and VBDs frequently target livestock and domestic animals, such as vaccination for RVF, brucellosis, rabies, or ectoparasite control for CCHF. Societal and stakeholder engagement underpins multisectoral approaches. Community engagement such as the NASA citizen science GLOBE (Global Learning and Observations to Benefit the Environment) program that use citizen-sourced imagery to identify the species and regions of mosquito prevalence.

Big data analytics can improve detection and control within and between countries irrespective of their development status. A 2020 study from Pakistan using big data analytics identified the need for localized containment activities and improved resource allocation [16]. Sentinel surveillance for priority pathogens could be candidates for initiating such efforts and achieving shared International Health Regulations approved in 2005 and global health security objectives.

Global and regional partnerships are critical to assure the technical and financial resources needed to supply vaccines and other medical countermeasures to control outbreaks in animals and humans. MENA is emerging as a hub in the pharmaceutical sector, driven by the pursuit of self-sufficiency in medicines and supported by the Gulf Cooperation Council (GCC) and regional collaboration. Greater cooperation with global stakeholders such as GAVI and CEPI, Nature4Health, Global Alliance for Veterinary Medicine, the Pandemic Fund and others can further empower this trend.

## Conclusions

MENA’s role as a nexus for movement of people and animals means that emerging human and animal disease threats in the region have global reach. Climate change and other factors, including many directly influenced by human activity, create, and amplify opportunities for a wide variety of pathogens to emerge or re-emerge or expand to new areas. The high degree of interconnectivity between the countries in MENA makes these partnerships and full implementation of One Health a priority to overcome the challenges facing these countries.

## Supplementary Material

ckae122_Supplementary_Data

## Data Availability

There are no data to share and make available for this manuscript.
