# Supplementary material for: Emerging and re-emerging disease threats in the Middle East and North Africa region—One Health approaches and potential strategies
Source: Eur J Public Health. 2025 Jan 13;35(Suppl 1):i3–5. doi: 10.1093/eurpub/ckae122 (PMC11725949; doi:10.1093/eurpub/ckae122)
Supplement: ckae122_Supplementary_Data [file ckae122_supplementary_data.docx]

Title: Emerging and re-emerging disease threats in the Middle East and North Africa region - One Health approaches and potential strategies

Authors: Sean V. Shadomy, Shahul H. Ebrahim, Sarah Anne J. Guagliardo, Liliana Sánchez-González, Kinda Zureick, Julie R. Sinclair, Dana A. Schneider, Allison T. Walker, Daniel C. Payne, Antonio R. Vieira, Kristin Heitzinger, Audrey Lenhart, Lisa P. Oakley, Jacob Clemente, Colin Basler, Charles B. Beard, Paige A. Armstrong, Heather Burke

*Introduction*

Emerging and re-emerging infectious diseases have no boundaries, and the Middle East and North Africa (MENA) region is no exception^[[1]](#footnote-2)^. Given the intricate mesh of commerce and livestock trade, mass gathering events, international travel, and population movement throughout the region, emerging health security threats in MENA can have global implications, and require a coordinated, One Health^[[2]](#footnote-3)^ approach across the human, animal, and environmental health sectors.

Emerging and re-emerging threats in MENA

Disease emergence events may result from evolution of existing organisms, the spread of known disease-causing agents into new populations or geographic areas , and the discovery of previously unrecognized pathogens. The emergence of Middle East Respiratory Syndrome coronavirus (MERS-CoV) in Jordan and Saudi Arabia in 2012 elevated MENA as a disease emergence hotspot, and, while earlier identification of agents such as the Alkhurma hemorrhagic virus in the 1990s gained little attention, the region has seen multiple pathogen emergence events during recent decades [1, 2]. Other recent emerging and re-emerging infections are detailed here and in Box 1.

Vector-borne diseases (VBD) in the region range from parasitic diseases such as malaria and leishmaniasis [3, 4], to arboviruses including *flaviviruses* (e.g., West Nile, dengue, Alkhurma hemorrhagic fever) [5-8], the *phlebovirus* Rift Valley Fever [9], and the *alphavirus* chikungunya [10]. A geographically diverse region, MENA countries have many arthropod disease vectors, including multiple species of mosquitoes and sandflies, and while they may be highly focal, increasing rainfall and water ponding can increase their distribution. *Aedes aegypti* and *Ae. albopictus* mosquitoes (vectors of dengue, Zika, chikungunya, and yellow fever viruses) have been detected in several countries [6]. Of particular concern is the establishment of the invasive mosquito *Anopheles stephensi* in the Horn of Africa and Yemen, threatening regional progress toward malaria elimination [11-13].

Many livestock and zoonotic diseases are widespread in the region, posing threats to health, food security, and trade despite control efforts. Foot and Mouth Disease, Peste des Petits Ruminants, and Rift Valley Fever (RVF) have all re-emerged in MENA [14]. Highly Pathogenic Avian Influenza (H5N1), first reported in Egypt in 2006 and Low Pathogenic Avian Influenza (H9N2) in Morocco in 2016, have been confirmed in wild and farmed birds in multiple countries and with associated zoonotic transmission [15, 16]. Rabies, bovine tuberculosis, leptospirosis, brucellosis, and parasitic zoonoses occur broadly across the region.

*Contributing factors, drivers and related issues:*

A variety of drivers and risk factors influence the emergence, geographic spread and burden of disease threats in MENA, including environmental, demographic, and host factors, and the increasing impact of antimicrobial resistance (Box 2). The effects of climate merit particular attention as it influences multiple other contributing factors. Climate change impacts emerging diseases pertinent to human and animal health through three interconnected processes: environmental change leading to cross-species transmission, global migration due to forced displacement, and urbanization. Carlson et al. predicted 4,000 new cross-species transmission events among wildlife by 2070 due to changes in climate and land use, increasing zoonotic pathogen spillover risk in heavily populated regions of Asia and Africa [17]. According to the World Bank, by 2050 climate change could result in the forced displacement of up to 18 million people in North Africa, Eastern Europe and Central Asia [18]. Most of those displaced will move into urban and peri-urban areas within the countries where they reside, exacerbating existing health challenges such as over-crowding, poverty, poor sanitation, and complex interactions between urbanization and infectious diseases [19].

Surveillance and serosurveys in human and animal populations are instrumental for detection and control, however these can be restricted or disrupted due to technical or resource constraints and conflicts (see Box 2), limiting the understanding of disease ecology and presence [20]. Furthermore, the paucity of MERS-CoV detections in Saudi Arabia and the occurrence of Crimean Congo Hemorrhagic Fever (CCHF) outbreaks in Iraq during the COVID-19 pandemic demonstrate how shifting attention to novel or high-visibility threats can interrupt existing disease surveillance and control programs [21, 22].

*Strategic One Health Approaches and Solutions:*

Strengthening human and animal health surveillance with established and innovative solutions can enable early detection and response to emerging threats. In 2021, the WHO Regional Committee for the Eastern Mediterranean endorsed a strategy for national integrated disease surveillance systems. By 2023, eleven countries were implementing event-based surveillance, exemplified by Qatar's use for early warning for the FIFA World Cup 2022 [23]. Resources supporting coordinated, One Health-based surveillance and information sharing are more readily available [24]. Incorporation of social and news media sources can improve alerting and mapping pathogen spread. Serosurveillance innovations such as multiplex serology can help identify anomalies suggesting pathogen emergence. Environmental surveillance such as wastewater sampling provides an additional tool for pathogen detection and monitoring.

Integrating travelers and other mobile populations into public health surveillance is critical given the population movement in the region and can enhance prevention of cross-border disease spread. Many countries focus efforts at official air, water, and ground points of entry (POE), which can detect and respond to overtly ill travelers, but may fail to detect infection in a- or pre-symptomatic travelers or those bypassing official POEs at porous border areas. Broader strategies that address health risks along the continuum of travel from point of origin to destination can include assessment of human and animal population mobility patterns and the connections between geographically separated communities to inform surveillance and effective information sharing between neighboring countries and across the region.

Incorporation of agent and genomic surveillance at POEs and by mass-gathering clinical services can detect infections in travelers and additionally inform emergence trends in their nations of origin. Such efforts were utilized in the US during COVID-19 [25] and routinely during the Hajj mass gatherings [26]. Countries with significant international connectivity can include the private sector in surveillance, such as travel clinics complementing early detection efforts and the provision of migrant health services. With support from U.S. CDC, countries in MENA are integrating travel medicine into national programs facilitating surveillance in international visitors and connecting to mobile populations [27].

Strengthening surveillance for animal health and zoonotic threats including on export and import can help protect economies, food safety and security and health security. Multiagent surveillance in imported livestock in Saudi Arabia has demonstrated the feasibility of such approaches [28]. It is important to include industry partners to ensure shared health security goals are met, and wild animal populations should be considered as important reservoirs. The Quadripartite^[[3]](#footnote-4)^ provides guidance and support to strengthen multisectoral preparedness, surveillance, and response, however these may be hindered by resource limitations, conflict or lack of political will. One Health collaborations are critical to success, and a key first step can be establishing regional platforms similar to WHO's Global Influenza Surveillance and Response System, or implementing collaborative approaches for specific threats such as the United Against Rabies “Zero by 30” to eliminate human deaths from canine rabies^[[4]](#footnote-5)^.

While two dengue vaccines (Dengvaxia and Qdenga), one chikungunya vaccine, and several prequalified yellow fever vaccines have been authorized for use in some countries, they are not widely available and may be subject to usage limitations [29-31]. VBD prevention and control therefore depends on integrated approaches. Integrated Vector Management (IVM) is a framework for vector control and surveillance emphasizing multisectoral participation that has been adopted by seven countries in MENA [32]; this could be expanded, perhaps first prioritizing countries with high VBD burden. With IVM strategies in mind, in 2021 CDC initiated the VecNet program to strengthen regional public health entomology networks. WHO-EMRO office organizes VecNet activities in MENA to strengthen national capacities to detect and respond to emerging threats including the spread of invasive vector species.

Campaigns to control zoonotic and VBDs frequently target livestock and domestic animals, such as vaccination for RVF, brucellosis, rabies, or ectoparasite control for CCHF. Such programs should clearly explain the benefits of control to help ensure uptake. Societal and stakeholder engagement underpins successful comprehensive multisectoral approaches. Community engagement can also contribute to scientific advances; an example is the NASA citizen science GLOBE (Global Learning and Observations to Benefit the Environment)^[[5]](#footnote-6)^ program using citizen-sourced imagery to develop artificial intelligence software to identify the species and regions of mosquito prevalence.

The interconnectedness between MENA countries provides them opportunities to partner and face shared challenges irrespective of their development status, and enhanced capacities such as big data analytics can improve detection and control within and between countries. A 2020 study from Pakistan using big data analytics identified the need for localized containment activities and improved resource allocation [33]. Sentinel surveillance for priority pathogens could be candidates for initiating such efforts and achieving shared International Health Regulations (2005) and global health security objectives.

Global and regional partnerships are critical to assure the technical and financial resources needed to supply vaccines and other medical countermeasures to control outbreaks in animals and humans. MENA is emerging as a hub in the pharmaceutical sector, driven by the pursuit of self-sufficiency in medicines and supported by the Gulf Cooperation Council (GCC) and regional collaboration. Greater cooperation with global stakeholders such as GAVI and CEPI, Nature4Health, Global Alliance for Veterinary Medicine, the Pandemic Fund and others can further empower this trend.

Conclusions

MENA’s role as a nexus for movement of people and animals means that emerging human and animal disease threats in the region have global reach. Climate change and other factors, including many directly influenced by human activity, create and amplify opportunities for a wide variety of pathogens to emerge or re-emerge or expand to new areas. The high degree of interconnectivity between the countries in MENA makes these partnerships and full implementation of integrative and collaborative concepts such as One Health a priority to overcome the challenges facing these countries.

| Box 1: Emerging and re-emerging infectious diseases of concern in humans and animals in the Middle East and North Africa Region  *Bacterial zoonoses*: *Leptospirosis is endemic in several countries including Egypt, Morocco, Tunisia, Algeria, Iran; *Brucellosis is a persistent problem in the region, with Syria, Iraq, Saudi Arabia, Turkey, and Iran having the world’s highest incidence rates [34]; anthrax remains an endemic threat in Turkey, Iran, and Pakistan; Plague remains a concern in Afghanistan; Bovine tuberculosis is reported throughout the region.  *Bacterial priority pathogens*: WHO recently updated their list of bacterial priority pathogens which pose the most significant public health threat and burden including critical antimicrobial resistant pathogens (carbapenem-resistant *Acinetobacter baumannii*, third-generation cephalosporin-resistant and/or carbapenem-resistant Enterobacterales, and rifampicin-resistant *Mycobacterium tuberculosis*) [35].  *Viral vector-borne illnesses*: *Chikungunya outbreaks have been reported in Djibouti, Pakistan, Sudan, and Yemen [10]; *Yellow Fever, and *West Nile Fever; locally-acquired *dengue has been reported in ten countries in the last decade, including recent outbreaks in Afghanistan, Egypt, Pakistan, Somalia, Sudan, and Yemen, and CDC currently classifies 11 countries in the region with either Frequent or Sporadic risk of dengue transmission (CDC, 2023); sandfly fever virus is reported in countries around the Mediterranean Sea and eastwards including Lebanon and Afghanistan.  *Viral zoonoses*: include *CCHF (CFR** up to 50%, reported in nine countries with prevalence in livestock ranging from 14%-29%) [34]; *MERS-CoV (CFR 27%-34%, ubiquitous seroprevalence (as high as 100%) among camels) [36]; *Rabies, *Rift Valley Fever; Q fever in Afghanistan and Iraq; Alkhurma virus in Saudi Arabia; zoonotic influenza viruses are reported in multiple countries in the region.  *Vaccine preventable childhood diseases*: These threats persist and outbreaks are seen in multiple countries in the region, including Diphtheria in Pakistan and Yemen; Measles and Polio in Afghanistan and Pakistan; and cholera in Somalia and Yemen.  *Parasitic infections*: *Malaria, *Leishmaniasis in Pakistan, Syria, Afghanistan; Tularemia has been reported to have seroprevalence of 7% in Jordan [37]; Endemic parasitic diseases (e.g., Echinococcus and Strongyloidiasis) are present in many countries.  *High-impact Transboundary Animal Diseases*: Foot and Mouth Disease persists in Algeria, Morocco has experienced a resurgence after 15 years; Morocco has experienced a resurgence of Peste des Petitis Ruminants after 7 years; *Rift Valley Fever  *Waterborne diseases*: Extensively drug-resistant typhoid fever has been reported in Pakistan; cholera reemergence or upsurge in Somalia, Yemen, Lebanon, Sudan, and Syria  **Included in the current list of priority Vector Borne and Zoonotic Disease by the WHO-EMRO: Brucellosis, CCHF, Chikungunya, Dengue, Leishmaniasis, Leptospirosis, Malaria, MERS-CoV, Rabies, Rift Valley Fever, West Nile Fever, Yellow Fever, Zoonotic influenza;*  ***CFR: case fatality rate* |
| --- |

| Box 2. Drivers influencing infectious disease emergence in the MENA region    *Conflicts*: In 2022, there were 58 conflicts in the region, of which 28 were violent crises and two full-scale wars, disrupting health systems and civilian infrastructure, inflicting numerous casualties, causing mass displacements of people and their animals, and creating multiple refugee crises [38].    *Population displacement*: In 2022, MENA accounted for one-fourth of the global internally displaced persons (16 million), making MENA the region with the second highest concentration of displaced persons following the sub-Saharan Africa region [39].  *High development disparity*: MENA has the world’s highest variation in GDP and per capita income among countries: (2022 GDP in USD: Qatar 87,662, Afghanistan 356) [40]. Life expectancy varies from 83 years in Israel to 64 in Yemen [41]. The human development index varies from 0.93 in UAE and Israel to 0.46 in Yemen [42].    *Climate change*: Being one of the most water-stressed and heat-stressed regions of the world, MENA is one of the world's most vulnerable to the impacts of climate change, enduring ever-higher temperatures, droughts, floods and other extreme weather events, increased water scarcity, all of which contribute to shifts in vector species and distribution. El Niño has affected 71% of drought years in the southern and southwestern parts of the Arabian Peninsula, and La Niña was linked to 38% of this area's flood years [43].  *Changing land and water use*: Anthropogenic activities such as deforestation, including the clearing of land for agricultural purposes and urbanization create habitats favorable for mosquito vectors, thus altering the risk of vector-borne disease. Urbanization adds to stress on water resources and contributes to prolonged heat exposure with associated health risks in both urban and rural settings.  *High burden of metabolic and other disorders:* Metabolic disorders have been associated to increased risk of complications and death in several infectious diseases, including dengue. The age-adjusted prevalence of diabetes reported in the MENA averaged 12.2% (range Kuwait, 19.6%, Yemen 11.3%) compared to 9.3% globally [44]. Obesity rates among men range from 2% to 55% in GCC countries [45]. High rates of vitamin D deficiency (60%- 80%) and hemoglobinopathies (thalassemia as high as 43%, sickle cell disease, 0.24%–5.8% , sickle cell trait 1.02%–45.8% ) also increase the risk for infections [46-48].  *Population movement across disease hotspot regions*: Foreign workforce accounts for about 80% of GCC workforce, the majority of whom are unskilled laborers originating from South and Southeast Asia [49]. GCC has emerged as a major international airline hub and tourist destination for the region (37% of arrivals in 2022), Asia (28%) and Africa (8%) [50].    *Mass gatherings:* MENA is home to the world’s largest annual international mass gathering involving over 180 countries (the Hajj), the largest domestic mass gatherings (Arbaeen), and a continuous year-round religious pilgrimage to these sites, all of which have been associated with disease transmission events. These mass gatherings attract elderly persons, and two-thirds of participants originate from and return to low to middle-income countries. Sports-related mass gatherings are on the rise in the GCC region.    *Changing animal contact patterns*: Changes in behaviors can result in increased direct or indirect contact with animals (livestock, wildlife, and exotic pets) and animal products. Increased contact with stray dogs and wildlife impacts human rabies case rates. Changes in consumption patterns such as of unpasteurized milk impacting rates of brucellosis, and increased risk for MERS-CoV outbreaks due to close contact with infected camels or consumption of raw camel products, are additional examples.  *Animal husbandry, legal and illegal movement of live animals*: Livestock contributes significantly to the GDP in many MENA countries (e.g., 40% of GDP in Somalia) [28]. Live animals are imported across MENA countries mostly for food and religious sacrifices but imports of those used in sporting events such as camel racing and as pets are increasing. MENA has additionally become a hotspot for wildlife trafficking as some airports in the region are among the main trafficking transit hubs worldwide [51].    *Sub-optimal human and animal healthcare infrastructure or coverage*: Countries with economic challenges and conflicts can have fragile healthcare systems, high disease burden, inadequate water and sanitation infrastructure, and low investment in disease detection, and prevention. In countries with significant international connectivity, host country health systems may not capture outbreaks among mobile populations as they may not seek care in the national health system.  *Antimicrobial resistance (AMR)*: Amidst many high-consequence pathogens, antimicrobial resistance (AMR) poses a growing threat to the health of humans, animals, plants, the environment, and the economy. Inappropriate antimicrobial use including overuse or other inappropriate prescribing practices, lack of access to appropriate treatment especially in conflict zones, insufficient infection prevention and control practices in clinical settings, and inadequate water, sanitation, and hygiene contribute to the spread and development of resistance in MENA. In MENA an estimated 255,676 deaths were associated with, and 68,292 deaths attributable to, bacterial AMR in 2019 (Institute for Health Metrics and Evaluation (IHME) 2024). That year, carbapenem-resistant *Acinetobacter* spp. accounted for 70.3% of patient bloodstream infections, higher than in the United States and European Union (Talaat 2022). Lack of coordinated, multisectoral approaches that include interoperable surveillance, laboratory networks, increased workforce development and capacity for high-quality infection prevention and control, antimicrobial stewardship, innovations in vaccines and therapeutics, and national action plans with sustainable funding will continue to increase the burden of AMR in the region. |
| --- |

References

1. Jones, K.E., et al., *Global trends in emerging infectious diseases.* Nature, 2008. **451**(7181): p. 990-993.

2. Stephens Patrick R., G.N., Schatz A. M., Schmidt J. P. and Drake John M. , *Characteristics of the 100 largest modern zoonotic disease outbreaks.* Phil. Trans. R. Soc. B, 2021. **376**.

3. Autino, B., et al., *Epidemiology of malaria in endemic areas.* Mediterr J Hematol Infect Dis, 2012. **4**(1): p. e2012060.

4. Du, R., et al., *Old World Cutaneous Leishmaniasis and Refugee Crises in the Middle East and North Africa.* PLOS Neglected Tropical Diseases, 2016. **10**(5): p. e0004545.

5. Eybpoosh, S., et al., *Epidemiology of West Nile Virus in the Eastern Mediterranean region: A systematic review.* PLOS Neglected Tropical Diseases, 2019. **13**(1): p. e0007081.

6. Humphrey, J.M., et al., *Dengue in the Middle East and North Africa: A Systematic Review.* PLOS Neglected Tropical Diseases, 2016. **10**(12): p. e0005194.

7. Madani, T.A., et al., *Alkhumra (Alkhurma) virus outbreak in Najran, Saudi Arabia: epidemiological, clinical, and laboratory characteristics.* J Infect, 2011. **62**(1): p. 67-76.

8. Gainor, E.M., E. Harris, and A.D. LaBeaud, *Uncovering the Burden of Dengue in Africa: Considerations on Magnitude, Misdiagnosis, and Ancestry.* Viruses, 2022. **14**(2).

9. Himeidan, Y.E., et al., *Recent outbreaks of rift valley Fever in East Africa and the middle East.* Front Public Health, 2014. **2**: p. 169.

10. Humphrey, J.M., et al., *Urban Chikungunya in the Middle East and North Africa: A systematic review.* PLoS Negl Trop Dis, 2017. **11**(6): p. e0005707.

11. Al-Eryani, S.M., et al., *Public health impact of the spread of Anopheles stephensi in the WHO Eastern Mediterranean Region countries in Horn of Africa and Yemen: need for integrated vector surveillance and control.* Malaria Journal, 2023. **22**(1): p. 187.

12. de Santi, V.P., et al., *Role of Anopheles stephensi Mosquitoes in Malaria Outbreak, Djibouti, 2019.* Emerg Infect Dis, 2021. **27**(6): p. 1697-1700.

13. Emiru, T., et al., *Evidence for a role of Anopheles stephensi in the spread of drug- and diagnosis-resistant malaria in Africa.* Nat Med, 2023. **29**(12): p. 3203-3211.

14. World Organisation for Animal Health. *World Animal Health Information System (WAHIS)*. 2024; Available from: <https://wahis.woah.org/#/home>.

15. El-Shesheny, R., et al., *H5 Influenza Viruses in Egypt.* Cold Spring Harb Perspect Med, 2021. **11**(6).

16. Almayahi, Z.K., et al., *First report of human infection with avian influenza A(H9N2) virus in Oman: The need for a One Health approach.* Int J Infect Dis, 2020. **91**: p. 169-173.

17. Carlson, C.J., et al., *Climate change increases cross-species viral transmission risk.* Nature, 2022. **607**(7919): p. 555-562.

18. Clement, V., Kanta Kumari Rigaud, Alex de Sherbinin, Bryan Jones, Susana Adamo, Jacob Schewe, Nian Sadiq, and Elham Shabahat, *Groundswell Part 2: Acting on Internal Climate Migration*. 2021, The World Bank: Washington, DC.

19. Baker, R.E., et al., *Infectious disease in an era of global change.* Nature Reviews Microbiology, 2022. **20**(4): p. 193-205.

20. Gayer, M., et al., *Conflict and emerging infectious diseases.* Emerg Infect Dis, 2007. **13**(11): p. 1625-31.

21. Alhilfi, R.A., et al., *Large outbreak of Crimean-Congo haemorrhagic fever in Iraq, 2022.* IJID Reg, 2023. **6**: p. 76-79.

22. Mallhi, T.H., et al., *Are we neglecting MERS-CoV during COVID-19 pandemic: a need for refocusing on surveillance of the deadly MERS-CoV infection.* Clin Microbiol Infect, 2024. **30**(2): p. 256-257.

23. Regional Committee for the Eastern Mediterranean, *Progress report on implementing the regional strategy for integrated disease surveillance: overcoming data fragmentation in the Eastern Mediterranean Region*. 2023, World Health Organization Regional Office for the Eastern Mediterranean.

24. World Health Organization. *OHHLEP inventory of One Health tools and resources*. 2023 15 November 2023; Available from: <https://www.who.int/publications/m/item/ohhlep-inventory-of-one-health-tools>.

25. Wegrzyn, R.D., et al., *Early Detection of Severe Acute Respiratory Syndrome Coronavirus 2 Variants Using Traveler-based Genomic Surveillance at 4 US Airports, September 2021-January 2022.* Clin Infect Dis, 2023. **76**(3): p. e540-e543.

26. Ebrahim, S.H., et al., *Pandemic H1N1 and the 2009 Hajj.* Science, 2009. **326**(5955): p. 938-940.

27. Ministry of Health, S.o.O. *Directorate General of Disease Surveillance and Control: Resources of Travel Medicine Service*. 2024 18 May 2024 14 May 2024]; Available from: <https://www.moh.gov.om/en/web/directorate-general-of-disease-surveillance-control/-1>.

28. Almasri, M., et al., *Hajj abattoirs in Makkah: risk of zoonotic infections among occupational workers.* Vet Med Sci, 2019. **5**(3): p. 428-434.

29. Paz-Bailey, G., et al., *Dengue.* The Lancet, 2024. **403**(10427): p. 667-682.

30. World Health Organization, *Yellow fever urban outbreak in Angola and the risk of extension.* Wkly Epidemiol Rec, 2016. **91**(14): p. 186-92.

31. U.S. Food and Drug Administration, *FDA Approves First Vaccine to Prevent Disease Caused by Chikungunya Virus*. 2023, U.S. Food and Drug Administration.

32. World Health Organization. *Integrated vector control management (IVM): Strengthening of national vector control capabilities in the Middle East and North Africa*. 2024 [cited 2024 5 April 2024]; Available from: <https://www.emro.who.int/malaria/gef-projects/integrated-vector-control-management.html>.

33. Abdur Rehman, N., et al., *Quantifying the localized relationship between vector containment activities and dengue incidence in a real-world setting: A spatial and time series modelling analysis based on geo-located data from Pakistan.* PLoS Negl Trop Dis, 2020. **14**(5): p. e0008273.

34. Bagheri Nejad, R., et al., *Brucellosis in the Middle East: Current situation and a pathway forward.* PLoS Negl Trop Dis, 2020. **14**(5): p. e0008071.

35. World Health Organization, *WHO Bacterial Priority Pathogens List, 2024: bacterial pathogens of public health importance to guide research, development and strategies to prevent and control antimicrobial resistance.* . 2024, World Health Organization: Geneva. p. 72.

36. Ebrahim SH, M.A., Kanagasabai U, Alfaraj SH, Alzahrani NA, Alqahtani SA, Assiri AM, Memish ZA. , *MERS-CoV Confirmation among 6,873 suspected persons and relevant Epidemiologic and Clinical Features, Saudi Arabia—2014 to 2019.* Lancet EClinicalMedicine, 2021. **41**.

37. Obaidat, M.M., et al., *Seroepidemiology, Spatial Distribution, and Risk Factors of Francisella tularensis in Jordan.* Am J Trop Med Hyg, 2020. **103**(2): p. 659-664.

38. International Institute for Strategic Studies (IISS). *The Armed Conflict Survey 2022: Middle East and North Africa Regional Analysis*. 2022 18 November 2022 [cited 2024 28 February 2024]; Available from: <https://www.iiss.org/online-analysis/online-analysis/2022/11/acs-2022-middle-east-and-north-africa/#:~:text=URL%3A%20https%3A%2F%2Fwww.iiss.org%2Fonline>.

39. IOM Global Data Institute, *Displacement Tracking Matrix: Middle East and North Africa — Quarterly Report October 1-December 31 2022 - Iraq, Libya, Sudan and Yemen*. p. 6.

40. The World Bank. *GDP per capita (current US$) - Middle East & North Africa, Sudan, Somalia, Afghanistan, Pakistan, West Bank and Gaza, South Sudan, Turkiye* 2024 [cited 2024 28 February 2024]; Available from: <https://data.worldbank.org/indicator/NY.GDP.PCAP.CD?locations=ZQ-SD-SO-AF-PK-PS-SS-TR>.

41. O'Neill, A. *Life expectancy at birth in the MENA countries 2021*. 2024 5 February 2024 [cited 2024 28 Febuary 2024]; Available from: <https://www.statista.com/statistics/804798/life-expectancy-at-birth-in-the-mena-countries/>.

42. The Global Economy. *Human development - Country rankings: Human Development Index (0 - 1), 2021*. 2024 [cited 2024 28 February 2024]; Available from: <https://www.theglobaleconomy.com/rankings/human_development/MENA/#:~:text=The%20average%20for%202021%20based,was%20in%20Yemen%3A%200.455%20points>.

43. Abid, M.A., et al., *ENSO relationship to summer rainfall variability and its potential predictability over Arabian Peninsula region.* npj Climate and Atmospheric Science, 2018. **1**(1): p. 20171.

44. El-Kebbi, I.M., et al., *Epidemiology of type 2 diabetes in the Middle East and North Africa: Challenges and call for action.* World J Diabetes, 2021. **12**(9): p. 1401-1425.

45. ALNohair S., *Obesity in gulf countries.* Int J Health Sci (Qassim), 2014. **8**(1): p. 79-83.

46. Abu-Shaheen, A., et al., *Epidemiology of Thalassemia in Gulf Cooperation Council Countries: A Systematic Review.* Biomed Res Int, 2020. **2020**: p. 1509501.

47. Abu-Shaheen, A., et al., *Sickle cell disease in gulf cooperation council countries: a systematic review.* Expert Rev Hematol, 2022. **15**(10): p. 893-909.

48. Lips, P., et al., *Current vitamin D status in European and Middle East countries and strategies to prevent vitamin D deficiency: a position statement of the European Calcified Tissue Society.* Eur J Endocrinol, 2019. **180**(4): p. P23-p54.

49. Alslan, H.K. *Dynamics of labor migration in the Gulf region*. 2022.

50. UN World Tourism Organization, *Tourism Grows 4% in 2021 but Remains Far Below Pre-Pandemic Levels*. 2022, UN World Tourism Organization,.

51. El Sayed, N. *Middle East major hub for wildlife trafficking*. Nature Middle East, 2018. DOI: doi:10.1038/nmiddleeast.2018.120.

1. For this discussion the authors define the Middle East and North Africa region as comprised of the following: countries: Afghanistan, Algeria, Bahrain, Djibouti, Egypt, Iran, Iraq, Israel, Jordan, Kuwait, Lebanon, Libya, Morocco, Oman, Pakistan, Palestine, Qatar, Saudi Arabia, Somalia, South Sudan, Sudan, Syria, Tunisia, Türkiye, United Arab Emirates, and Yemen. [↑](#footnote-ref-2)
2. https://www.who.int/publications/m/item/one-health-definitions-and-principles [↑](#footnote-ref-3)
3. The Food and Agriculture Organization of the United Nations (FAO), World Health Organization (WHO), World Organisation for Animal Health (WOAH) and United Nations Environment Programme (UNEP) [↑](#footnote-ref-4)
4. https://www.unitedagainstrabies.org/ [↑](#footnote-ref-5)
5. https://www.globe.gov/ [↑](#footnote-ref-6)
